# Prevalence and associated factors of fear of childbirth among pregnant women in Saudi Arabia

**DOI:** 10.3389/fpsyt.2025.1715407

**Published:** 2026-01-07

**Authors:** Deemah Alateeq, Nujud bin Khunayn, Rayouf Alqahtani, Shaden Alqahtani, Khawlah Alharbi, Sarah Aldossary, Aljouhara Alaasmi, Rawan Alanazi, Nouran Aleyeidi, Amel Fayed

**Affiliations:** 1 Internal Medicine Department, College of Medicine, Princess Nourah bint Abdulrahman University, Riyadh, Saudi Arabia; 2 Community Medicine Department, College of Medicine, Princess Nourah bint Abdulrahman University, Riyadh, Saudi Arabia

**Keywords:** anxiety, depression, fear of childbirth, pregnancy, tokophobia

## Abstract

**Background:**

Fear of childbirth (FOC) is a common concern among pregnant women and can significantly impact maternal and fetal well-being. This study estimates the prevalence of childbirth fear, its determinants and correlation with depression and anxiety, and related factors among pregnant women in Saudi Arabia.

**Methods:**

The study conducted a cross-sectional online survey of a convenience sample of 617 pregnant women in Saudi Arabia using a self-administered questionnaire collected over one month. The questionnaire included the Wijma Delivery Expectancy Questionnaire (W-DEQ) for FOC and the Generalized Anxiety Disorder Scale (GAD-7) and Patient Health Questionnaire (PHQ-9) for anxiety and depression.

**Results:**

The prevalence of high FOC was 7%. Significant associations were found between FOC and many variables, including low income (p=0.001), unstable marital relationships (p=0.001), first-trimester gestational age (p=0.04), lack of follow-up with pregnancy (p=0.001), and following up in both private and government hospitals (p=0.02). There was a weak positive correlation between FOC and anxiety, as well as depressive symptoms.

**Conclusion:**

While FOC is a notable concern, it is not pervasive among pregnant women in Saudi Arabia. Targeted psychosocial interventions for vulnerable and at-risk groups, together with improved continuity of antenatal care, are recommended, and future studies should aim to represent all regions of the country.

## Introduction

Women during pregnancy are highly susceptible to psychological health issues. The mental health problems that are most commonly associated with pregnancy are depression and anxiety (1). It’s found that abortions, marital issues, an unhealthy work-life balance, excessive stress, physical conditions, and other comorbidities can all be linked to mental health issues among pregnant women (1). A study conducted in KSA on pregnant women attending clinics showed that 26.8% were depressed while 23.6% were anxious (2).

Women’s mental health during pregnancy has significant implications for the mother’s well-being and her unborn child’s development, health, and well-being (3, 4). Even after controlling the effects of established sociodemographic, obstetric, and behavioral risk factors, a growing body of empirical evidence from population-based studies suggests two indicators of women’s mental health during pregnancy. The two indicators are psychosocial stress and social support, which may significantly influence fetal development and infant birth outcomes, such as birth weight and gestation length (4).

Fear of childbirth (FOC), known as tokophobia, is an issue seen in nulliparous, primiparous, and multiparous women with adverse health consequences and effects on labor, delivery, and the postpartum period (5). The fear of giving birth is commonly conceived as an anxiety-related experience. Clinical presentations of giving birth fear are frequently characterized by symptoms similar to those of various emotional disturbances (6). Women’s internal, external, and personal conditions, such as poor mental health, anxiety disorders, prior trauma, and social circumstances, can contribute significantly to FOC (5).

Using the Wijma delivery expectancy/experience questionnaire (W-DEQ), a study involving 125 pregnant women from Brazil showed that 12% had FOC associated with anxiety and depression (7). Moreover, A study conducted in Karnataka with 388 pregnant women showed that 45.4% feared childbirth (8). Fear of delivery was substantially correlated with teenage pregnancy, nulliparity, primigravida status, and abortion (8). In addition, a study in China showed a negative link between FOC and social support and a positive correlation with pregnancy-related stress and depressive symptoms (9).

Furthermore, numerous studies conducted in the Middle East showed some pregnant women suffer from FOC. A study in Yemen revealed that (72%) of pregnant women had FOC (10). Another study done in Egypt among pregnant women showed that about half of the participants (55.33%) had increased levels of fear related to childbirth (11). Moreover, a study on factors contributing to the fear of childbirth among pregnant women in Iran showed that (89.3%) of the participants expressed worry about childbirth (12). However, no study evaluated the prevalence and associated factors of fear of childbirth among pregnant women in Saudi Arabia. This study aims to estimate the prevalence of childbirth fear, its determinants, and its correlation with depression and anxiety among pregnant women in Saudi Arabia.

## Methods

This observational, cross-sectional online survey included Saudi pregnant women aged 18–46 years and excluded women younger than 18 or older than 46. The study was conducted in Saudi Arabia between December 2023 and April 2024 using an online questionnaire on RedCap, distributed via social media platforms. A non-probability snowball convenience sampling strategy was adopted, as no national sampling frame of pregnant women was available, and an online design was considered the most feasible way to reach women from different regions within the short study period and with the available resources. Saudi pregnant women aged 18–46 were included, whereas women under 18 or over 46 were excluded. The survey link was initially disseminated by the study investigators through social media applications (e.g., WhatsApp and X/Twitter), perinatal support groups, and OB/GYN clinics at King Abdullah bin Abdulaziz University Hospital (KAAUH). Participants were encouraged to forward the link within their networks, creating a snowball effect. Because the link was shared across multiple platforms and personal networks, it was not possible to determine the exact number of women who viewed the invitation, and a formal response rate could not be calculated. Ethical approval was taken from the Institutional Review Board (IRB) at Princess Nourah bint Abdulrahman (PNU) (IRB Log Number: 23-0855). Every person has the right to accept or refuse to participate in the study. Participants did not require clarification of their identities, and the data remain confidential and are used only for the study. Consent was obtained from participants before their participation.

### Sample size

The required sample size was calculated using a single-proportion formula for prevalence studies below:


$$\left[\text{n}={\text{Z}}^{\;2}\text{ p}\frac{\left(1-\text{p}\right)}{{\text{d}}^{\;2}}\right]$$

n is the required sample size, Z is the standard normal deviate corresponding to the desired confidence level, p is the expected prevalence of fear of childbirth, and d is the acceptable margin of error. We assumed a 95% confidence level (*Z* = 1.96) and a precision (*d*) of approximately 3%. The expected prevalence (*p*) of fear of childbirth was not arbitrarily chosen, but informed by previous studies from similar settings, which reported prevalence estimates ranging from about 6.3% to 14.8%. To obtain a conservative estimate and maximize statistical power, we used a prevalence near the upper end of this range (p ≈ 0.15), which yielded a minimum required sample of approximately 540 participants. This figure was then rounded up to a target minimum of 560 pregnant women to allow for some incomplete or ineligible responses in the online survey. Data collection continued throughout the predefined study period, and all eligible women who completed the questionnaire during this time were included in the analysis, resulting in a final analytic sample of 617 participants.

### Measures

The questionnaire consisted of four main components. The first component collected sociodemographic and background information, including age, region of current residency, marital status, living situation, education level, employment status, and household income, as well as selected medical and obstetric history variables. Household income was recorded initially using three self-reported categories (“enough with saving”, “enough”, and “not enough”). For some analyses, “not enough” was treated as an indicator of low income, while “enough” and “enough with saving” were combined and interpreted as higher income; this re-coding is indicated where relevant in the Results and Discussion.

### Fear of childbirth

Fear of childbirth was assessed based on the Arabic version of the W-DEQ Wijma Delivery Expectancy/Experience Questionnaire (W-DEQ), which has 10 questions based on the original 33-item version (13). Each item reflects cognitive and emotional expectations about labor and birth (such as perceived control, safety, and anticipated pain). Items are scored on a 3-point Likert scale from 1 to 3, producing a total score between 10 and 30, with higher scores indicating greater fear of childbirth. The Arabic 10-item version was previously used among pregnant women in Egypt and showed good internal consistency, supporting its use in Arabic-speaking antenatal populations (11). In line with that study, and to facilitate comparison, we categorized W-DEQ scores into three groups: no fear (score of 10), low fear (scores 11-20), and high fear (scores >20). Although the short form has demonstrated acceptable internal consistency, detailed psychometric evaluation in Saudi pregnant women is still limited, and this should be considered when interpreting the findings.

### Anxiety and depression

Anxiety symptoms during the previous four weeks were measured using the Generalized Anxiety Disorder 7-item scale (GAD-7). The Arabic version, validated in a Saudi sample and shown to have good reliability and validity, was used (14). The GAD-7 consists of 7 items, each rated from 0 (not at all) to 3 (nearly every day), yielding a total score of 0 to 21. Scores are typically categorized as minimal anxiety (<5), mild anxiety (5-9), moderate anxiety (10-14), and severe anxiety (≥15). Depressive symptoms during the previous two weeks were assessed using the Patient Health Questionnaire-9 (PHQ-9), for which the Arabic version validated in Saudi Arabia was also used (14). The PHQ-9 includes nine items, each scored from 0 to 3, yielding a total score from 0 to 27, categorized as minimal depression (<5), mild depression (5-9), moderate depression (10-14), moderately severe depression (15-19), and severe depression (20-27).

### Statistical analysis

Data were analyzed using IBM SPSS Statistics Version 26. Descriptive statistics (means, standard deviations, frequencies, and percentages) summarized sociodemographic, obstetric, and psychological variables. Normality tests assessed whether the continuous variables were normally distributed. Normally distributed variables were summarized by means and standard deviations, while skewed count variables were analyzed using medians and interquartile ranges in the supporting analyses. For bivariate analyses, chi-square tests evaluated associations between categories of fear of childbirth (no, low, high) and categorical sociodemographic or obstetric variables, with Fisher’s exact test applied when expected cell counts were small. For continuous variables, independent-samples t tests were used when normality was reasonable, and non-parametric tests (Mann-Whitney U or Kruskal-Wallis) were applied for skewed distributions. Continuous W-DEQ, GAD-7, and PHQ-9 scores were correlated using Spearman’s rank correlation coefficients due to their skewed distributions. All hypothesis tests were conducted at a significance level of p < 0.05. Because of the cross-sectional, exploratory design and the relatively small number of women with high fear of childbirth, no multivariable regression models were fitted; the study focused on describing prevalence and bivariate associations rather than estimating fully adjusted effect sizes.

## Results

### Socio-demographic data, medical, and psychiatric history

A total of 617 eligible pregnant women completed the questionnaire and were included in the analysis, which exceeded the minimum required sample size of 560. The sociodemographic characteristics of the sample are presented in **Table 1**. The mean age of the participants in this study was approximately 31 years. The majority were residents of the middle region (68.7%), had a bachelor’s degree (75.7%), lived with their husbands (90.9%), had a marital period of more than three years (65.6%), and had a stable marital relationship without problems (79.4%). Almost half were housewives (48.9%). Most women reported that their household income was either “enough” (54.8%) or “enough with saving” (33.5%), whereas 11.7% reported that their income was “not enough”; in subsequent analyses, this latter group is described as having low income. The majority did not have chronic illnesses (88.2%) or any psychological disturbances (93.8%).

**Table 1:** Socio-demographic characteristics, medical, and psychiatric of the participants (n=617).

Variables	n (%)
Age	31.47 + 7.5
Residency	
Middle region	424 (68.7%)
Eastern region	54 (8.8%)
Northern region	84 (13.6%)
Western region	30 (4.9%)
Southern region	25 (4.1%)
Education	
School	85 (13.8%)
University	467 (75.7%)
Post-graduate	62 (10%)
Living situation	
Living with husband	561 (90.9%)
Living alone	12 (1.9%)
Living with family	44 (7.1%)
Marital period	
Less than year	79 (12.8%)
One to three years	132 (21.4%)
More than three years	405 (65.6%)
Occupation	
Employed	248 (40.2%)
Housewife	302 (48.9%)
Student	67 (10.9%)
Income	
Enough with saving	207 (33.5%)
Enough	338 (54.8%)
Not enough	72 (11.7%)
Nature of marital relationship	
Stable without problems	490 (79.4%)
Stable with problems	100 (16.2%)
Unstable	23 (3.7%)
Diagnosed with chronic illness	
Yes	73 (11.8%)
Diagnosed with psychological disturbances	
Yes	37 (6%)

### Obstetric history

Regarding the history of pregnancy and labor, as shown in **Table 2**, most participants’ current pregnancies were their third or higher (46%). Counts of previous obstetric events were generally low: just over one-third of women were primigravida, and nearly one-half reported at least one prior pregnancy. Because the distributions of the number of deliveries, abortions, and living children were skewed, these variables were recorded into ordered categories (0, 1-2, >3) and are presented in **Table 2** as counts and percentages. The majority had a natural vaginal delivery (43.4%), reported no previous complications during labor (81%), and were currently in the third trimester, with a gestational age of 7 months or more (43.6%). Most participants reported no previous complications during pregnancy (87.8%), attended antenatal care regularly (77.8%), and most commonly received follow-up in government hospitals (36.6%), followed by private hospitals (36.1%) and both sectors (22.5%).

**Table 2:** Obstetric characteristics of the study population (n=617).

Variables	n (%)
Number of pregnancies	
The current is the first	214 (35.5%)
The current is the second	106 (17.6%)
The current is the third or more	277 (46%)
Number of deliveries	
0	187 (30.3%)
1-2	126 (20.4%)
≥3	304 (49.3%)
Number of abortions	
0	367 (59.5%)
1-2	230 (37.3%)
≥3	20 (3.2%)
Number of children	
1	214 (34.7%)
1-2	113 (18.3%)
≥3	290 (47.0%)
Nature of previous birth	
Natural	268 (43.4%)
Cesarean section	94 (15.2%)
Both	63 (10.2%)
No history of delivery	187 (30.3%)
Complications of previous labor	
Yes	95 (15.4%)
Current gestational age	
First trimester	167 (27.1%)
Second trimester	156 (25.3%)
Third trimester	269 (43.6%)
Pregnancy complications	
Yes	68 (11%)
Pattern of medical follow-up	
Regularly	480 (77.8%)
Sometimes	92 (14.9%)
Rarely	17 (2.8%)
Never	15 (2.4%)
Place of follow-up	
Private hospital	223 (36.1%)
Government hospital	226 (36.6%)
Both	139 (22.5%)
Other	18 (2.9%)

### Prevalence of fear of childbirth and its associates

**Figure 1:**
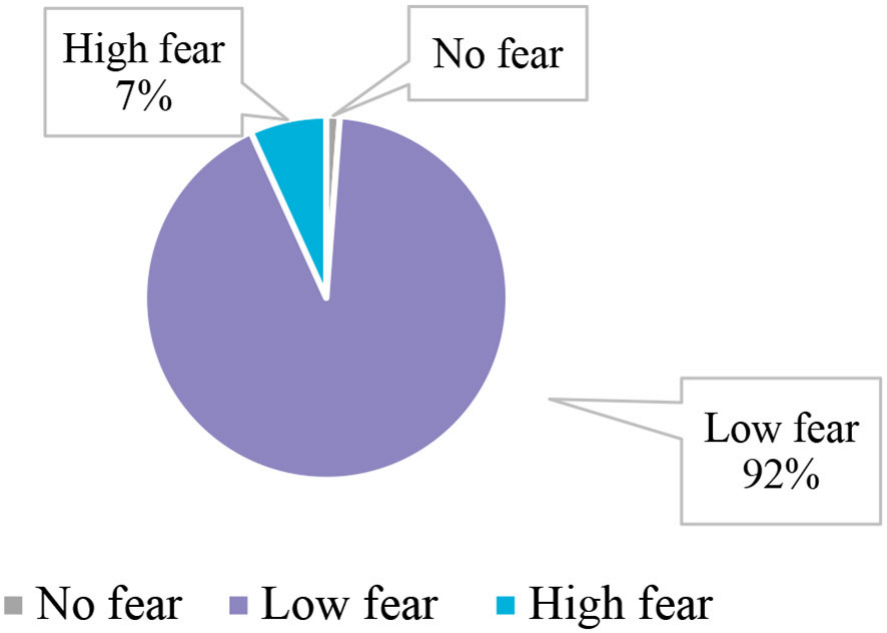
The prevalence of FOC among pregnant women (n=617).

As shown in **Figure 1**, according to the categorized W-DEQ short-form scores, 7% of participants had high levels of fear of childbirth, while 92% had low levels, and only 1% had no fear of childbirth. As shown in **Table 3**, FOC was significantly associated with low income (i.e. women who reported that their income was not enough; p = 0.001), unstable marital relationships (p = 0.001), and first-trimester gestational age (p = 0.04). Moreover, there was a significant relation between FOC and pregnant women who never followed up with their pregnancy (p=0.001) and pregnant women who followed up in both private and government hospitals (p=0.02).

**Table 3:** Relation between FOC and the associated factors.

Variables		WEJMA screening tool Results, n (%)			P value
		No fear	Low Fear	High Fear	
Residency	top region	3 (0.7%)	378 (91.7%)	31 (7.5%)	0.071
	Eastern region	0 (0%)	52 (100%)	0 (0%)	
	Northern region	3 (3.7%)	75 (91.5%)	4 (4.9%)	
	Western region	0 (0%)	26 (86.7%)	4 (13.3%)	
	Southern region	1 (4%)	22 (88%)	2 (8%)	
Education	School	1 (1.2%)	80 (95.2%)	3 (3.6%)	0.641
	University	6 (1.3%)	413 (91.4%)	33 (7.3%)	
	Post-graduate	0 (0%)	57 (91.9%)	5 (8.1%)	
Living situation	Living with husband	5 (0.9%)	507 (92.5%)	36 (6.6%)	0.053
	Living alone	1 (9.1%)	10 (90.9%)	0 (0%)	
	Living with family	1 (2.4%)	36 (85.7%)	5 (11.9%)	
Marital period	Less than year	3 (3.8%)	70 (89.7%)	5 (6.4%)	0.230
	One to three years	1 (0.8%)	118 (92.2%)	9 (7%)	
	More than three years	3 (0.8%)	365 (92.4%)	27 (6.8%)	
Occupation	Employed	3 (1.2%)	222 (91%)	19 (7.8%)	0.527
	Housewife	2 (0.7%)	271 (93.1%)	18 (6.2%)	
	Student	2 (3%)	60 (90.9%)	4 (6.1%)	
Income *	Enough with saving	4 (2%)	189 (93.1%)	10 (4.9%)	0.001
	Enough	3 (0.9%)	308 (93.9%)	17 (5.2%)	
	Not enough	0 (0%)	56 (80%)	14 (20%)	
Nature of marital relationship *	Stable without problems	6 (1.2%)	457 (94.8%)	19 (3.9%)	0.001
	Stable with problems	0 (0%)	78 (81.3%)	18 (18.8%)	
	Unstable	1 (5%)	15 (75%)	4 (20%)	
Diagnosed with chronic illness	Yes	0 (0%)	66 (91.7%)	6 (8.3%)	0.541
	No	7 (1.3%)	487 (92.1%)	35 (6.6%)	
Diagnosed with psychological disturbance	Yes	1 (2.9%)	31 (88.6%)	3 (8.6%)	0.571
	No	6 (1.1%)	521 (92.2%)	38 (6.7%)	
Number of pregnancies	The current is the first	4 (1.9%)	199 (93%)	11 (5.1%)	0.360
	The current is the second	0 (0%)	95 (90.5%)	10 (9.5%)	
	The current is the third	3 (1.1%)	257 (92.8%)	17 (6.1%)	
Number of deliverers	Primipara	4 (57.1%)	187 (34.4%)	10 (25%)	0.394
	Multipara	3 (42.9%)	277 (51%)	22 (55%)	
	Grand Multipara	0 (0%)	79 (14.5%)	8 (20%)	
Number of abortions	No history	7 (100%)	366 (68%)	24 (60%)	0.166
	Once or twice	0 (0%)	141 (26.2%)	11 (27.5%)	
	3 or more	0 (0%)	31 (5.8%)	5 (12.5%)	
Nature of previous birth	Natural	3 (1.1%)	243 (92%)	18 (6.8%)	0.432
	Cesarean section	0 (0%)	82 (90.1%)	9 (9.9%)	
	Both	2 (3.2%)	55 (88.7%)	5 (8.1%)	
	No history of delivery	2 (1.1%)	173 (94%)	9 (4.9%)	
Complications of previous labor	Yes	2 (2.2%)	77 (85.6%)	11 (12.2%)	0.065
	No	5 (1%)	459 (92.9%)	30 (6.1%)	
Current gestational age *	One to three	4 (2.4%)	143 (86.7%)	18 (10.9%)	0.040
	Four to six	1 (0.7%)	145 (94.8%)	7 (4.6%)	
	Seven or more	2 (0.7%)	253 (94.1%)	14 (5.2%)	
Pregnancy complications	Yes	1 (1.5%)	57 (85.1%)	9 (13.4%)	0.071
	No	6 (1.1%)	496 (92.9%)	32 (6%)	
Pattern of medical follow up *	Regularly	3 (0.6%)	447 (93.7%)	27 (5.7%)	0.001
	Sometimes	0 (0%)	79 (88.8%)	10 (11.2%)	
	Rarely	1 (5.9%)	15 (88.2%)	1 (5.9%)	
	Never	3 (20%)	10 (66.7%)	2 (13.3%)	
Place of follow up *	Private hospital	3 (1.4%)	209 (94.1%)	10 (4.5%)	0.020
	Government hospital	3 (1.3%)	210 (93.3%)	12 (5.3%)	
	Both	0 (0%)	119 (87.5%)	17 (12.5%)	
	Other	1 (5.9%)	14 (82.4%)	2 (11.8%)	

*Indicates significant results.

### Fear of childbirth, anxiety, and depression

**Figure 2:**
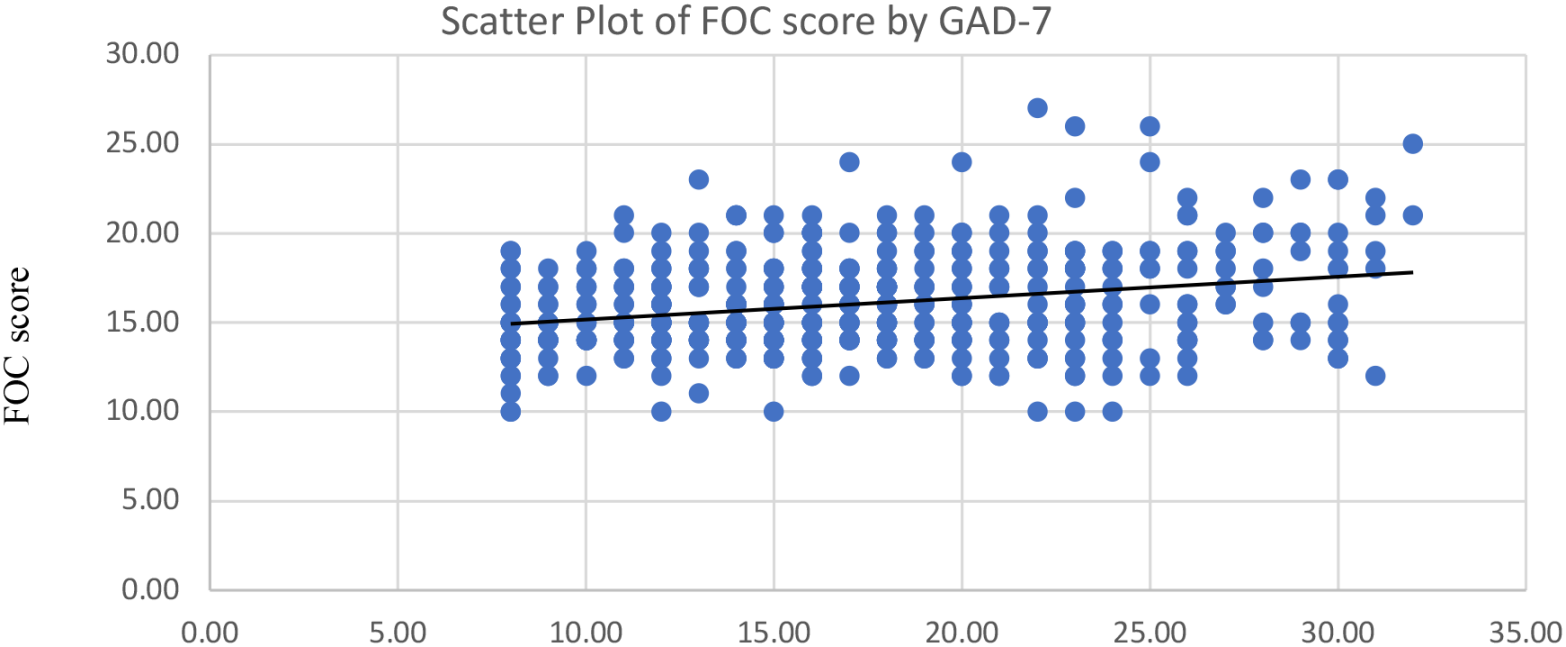
Correlation between fear of childbirth (FOC) and Anxiety (GAD-7).

**Figure 3:**
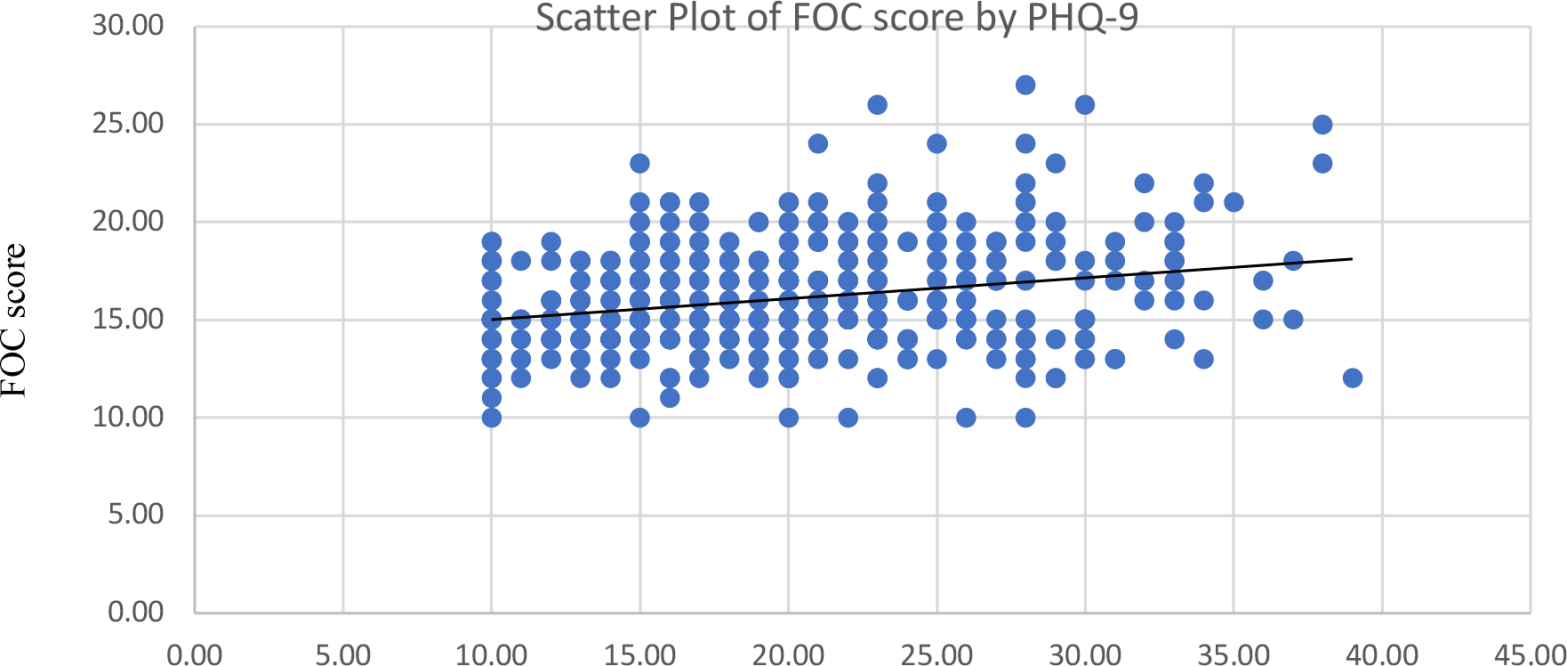
Correlation between fear of childbirth (FOC) and Depression (PHQ-9).

Among the participants, 66% had severe anxiety, 25% had moderate anxiety, and 9% had mild anxiety. The prevalence of severe (38%) and moderately severe (38%) depression were equally high among the participants, and 24% had moderate depression on the PHQ-9. Using Spearman’s rank correlation, we observed weak positive associations between continuous W-DEQ scores and GAD-7 anxiety scores, and between W-DEQ scores and PHQ-9 depression scores; these correlations were small in magnitude and not statistically significant (**Figures 2**, **3**).

## Discussion

Fear of childbirth is a neglected health dilemma in most developing countries (15). To our knowledge, this is the first study to estimate and comprehensively investigate the prevalence of fear of childbirth, its associated factors, and its correlation with anxiety and depression among pregnant women in Saudi Arabia. In this cross-sectional online survey, 7% of participants met the threshold for high fear of childbirth (FOC) on the 10-item Arabic short form of the Wijma Delivery Expectancy Questionnaire (W-DEQ), while the vast majority reported low fear. This estimate appears to fall within the lower range of international prevalence figures; however, direct comparisons with studies from the United States, European countries, and Australia should be interpreted with caution, as those studies used the original 33-item W-DEQ with different response scales and cut-off values. In this context, a study conducted in the United States showed that 7.7% of pregnant women reported a serious fear of childbirth (16), a study in six European countries reported that 11% had a severe fear of childbirth (17), and an Australian study reported that 4.8% of pregnant women had a severe fear of childbirth (18). These figures provide useful background but are not strictly comparable to the present findings because of the differences in the version and scoring of the W-DEQ.

Moreover, the result of this study is lower than the findings reported in Brazil, Egypt, and Turkey, which might be due to the majority of participants having a high educational level, enough income, and stable relationships without problems. It is plausible that this relatively advantaged profile, combined with regular antenatal follow-up for most women in the sample, contributed to the lower observed prevalence of high FOC. A Brazilian study at a maternity hospital in southern Brazil reported a prevalence of 12% (7). An Egyptian study conducted in an antenatal clinic showed that 55.33% of the participants had a fear of childbirth (11). A Turkish study showed that 42.4% of pregnant women had a fear of childbirth associated with low education levels, unemployment status, and low financial status (19). These comparisons collectively indicate that social and economic resources, educational achievement, and access to structured antenatal care might help cushion women against more extreme forms of childbirth fear, but cultural variations in the articulation and disclosure of FOC might also be relevant.

This study found a significant association between FOC and low income. Women with low household incomes experienced higher levels of FOC, a trend also reported in previous studies conducted in Iran (12, 20). Financial strain can increase anxiety about how to cover costs of pregnancy care, delivery, and the postnatal process, and furthermore, it may enhance concerns about complications and the possibility of receiving professional help in time.

An unstable relationship with the husband was found to be associated significantly with increased levels of FOC, reinforcing the importance of marital support. This relationship between marital status and FOC has been corroborated by a study conducted in Turkey, which indicated that poor spouse support elevates the fear of childbirth (21). Women who described their relationship as unstable may anticipate limited emotional and practical support during labor and the postpartum period, which can intensify perceptions of vulnerability and fear.

Furthermore, women in their first trimester exhibited higher levels of FOC, contrasting with results from a study conducted in Ethiopia, where FOC was reportedly lower in the early stages of pregnancy (22). This might be because women in the third trimester were more prepared for childbirth than those in the first. Early in pregnancy, women may have less information about labor and delivery, fewer opportunities to discuss concerns with healthcare providers, and a longer period of uncertainty about pregnancy outcomes, all of which may elevate fear. In contrast, as gestation advances, repeated contact with antenatal services and greater exposure to educational messages may help some women to reframe or manage their fears.

In addition, women who did not attend any prenatal care appointments or who attended appointments at both governmental and private hospitals showed high levels of FOC. This finding underscores the critical role of consistent and continuous prenatal care in alleviating fear and anxiety related to childbirth. Regular and trusted medical follow-up provides reassurance and valuable information to expectant mothers, reducing uncertainty and fear. Women who move between different providers or sectors may experience fragmented care and conflicting messages about pregnancy and birth, which could undermine trust and increase apprehension. Consequently, enhancing access to and continuity of prenatal care could be a key strategy in addressing and mitigating the fear of childbirth among pregnant women. Interventions that strengthen ongoing relationships with a small number of trusted providers, provide structured antenatal education, and screen routinely for high FOC may be particularly valuable in this setting. Further research is needed to explore these associations in greater depth and develop tailored interventions to reduce FOC and improve overall maternal well-being.

This study revealed weak positive correlations between manifesting depressive and anxiety symptoms and fear of childbirth. However, they were not statistically significant. These findings align with a Brazilian study that also demonstrated weak positive correlations between FOC and depression and anxiety, thus supporting our results (7). In the present analysis, non-parametric Spearman correlation coefficients were used because the distributions of W-DEQ, GAD-7, and PHQ-9 scores were skewed, and the small effect sizes suggest that, in this sample, FOC and general symptoms of anxiety and depression only partially overlapped. Although the correlations in our study were weak and not statistically significant, they suggest a potential relationship that warrants further investigation with a larger sample size to understand the dynamics between depression, anxiety, and fear of childbirth. These correlations reflect internal associations within this sample based on the 10-item Arabic W-DEQ short form, and the interpretation of absolute FOC severity levels should be viewed in light of this measurement approach. Future studies that employ the full 33-item W-DEQ or formally cross validate the short form against the original scale would help to determine whether similar patterns of association with anxiety and depression are observed. Such insights could be crucial for developing targeted interventions to alleviate depression and anxiety in expectant mothers, ultimately contributing to better maternal and fetal outcomes.

Although this study is the first to assess FOC in SA, it has several limitations. First, fear of childbirth was measured using the 10-item Arabic short form of the W-DEQ, which differs in length, response scale, and cut-off values from the original 33-item W-DEQ widely used in international research. These differences may affect the estimated prevalence of high FOC and limit the comparability of the present findings with studies based on the full instrument, so the results should be interpreted with caution and regarded as preliminary until further psychometric work cross validates the short form against the standard scale. Moreover, the available validation work for the Arabic short form is limited to one Egyptian study, so a more detailed psychometric evaluation in Saudi populations is needed. Second, the majority of participants in the study were from the middle region of Saudi Arabia (68.7%), which may not fully represent the experiences and prevalence of FOC in other regions of the country. Third, recruitment relied on an online, non-probability snowball convenience sample distributed through social media and a single university hospital, which is likely to have favored women with internet access, and higher education (5, 15). This creates a selection bias and restricts the extrapolation of the results to all pregnant women in Saudi Arabia, especially to those who have limited access to digital technology. Since the survey link was sent via social networks and support groups, the population of women to whom invitations were sent could not be known, and a response rate could not be calculated, increasing the risk of non-response bias. Fourth, the cross-sectional design does not allow making any causal assumptions regarding the direction of the associations between FOC, income, marital relationship, antenatal care, and mental health symptoms (4, 5). Lastly, the test was based on bivariate comparisons and simple correlations; the number of women with high FOC was too low to establish reliable multivariate regression models, and therefore, it is not possible to rule out residual confounding by unmeasured or correlated factors.

Despite these limitations, the study offers valuable preliminary evidence about the burden and correlates of FOC in pregnant women in Saudi Arabia and identifies some of the modifiable social and healthcare determinants that can be addressed in clinical practice (15, 22). Psychosocial support of women with financial constraints or with an unstable family, enhancing the quality and continuity of antenatal care, and regularly measuring childbirth fear and anxiety and depression can be used to ameliorate maternal mental health (5, 7). Future research should employ probability-based sampling in community and clinic settings, include women from all regions and a wider range of healthcare facilities, and incorporate robust multivariable modelling to clarify the independent contribution of social, obstetric, and psychological factors to FOC.

## Conclusions

In conclusion, while fear of childbirth (FOC) exists among participants, the majority experience it at a low level, highlighting its presence as a notable but not pervasive concern with a weak positive correlation between anxiety and depression. Psychosocial intervention for FOC among vulnerable populations such as women with low-income and unstable marriages might be helpful. Regular and continuous prenatal care might also be beneficial in alleviating FOC and designing and applying antenatal education classes for those who have a higher risk of having a fear of childbirth and who have psychiatric disorders. In addition, more attention should be paid to the mental health of pregnant women in first-trimester gestational age.

These findings underscore the complex interplay of individual and environmental factors in shaping maternal mental health during pregnancy. Given the complex nature of FOC and its potential impact on maternal well-being, it is imperative to conduct further research on this topic, particularly in Saudi Arabia, to better understand its prevalence, determinants, and implications for maternal health outcomes. Additionally, efforts should be made to educate and support pregnant women about FOC, equipping them with the necessary resources and coping strategies to navigate this aspect of pregnancy more effectively. By addressing these gaps in knowledge and support, we can strive towards improving the overall maternity care and mental health support for pregnant women in Saudi Arabia. Further research is needed to study which other psychiatric illnesses that could affect pregnant women’s fear of childbirth and to study the association between anxiety and depression and associated factors that affect fear of childbirth in Saudi Arabia. Future initiatives should focus on representing different settings and all regions of Saudi Arabia.

## Data Availability

The raw data supporting the conclusions of this article will be made available by the authors, without undue reservation.
